# Functional Outcomes of Emergency Surgery for Perforated Diverticulitis, Hinchey Grade III

**DOI:** 10.1007/s00268-023-06961-2

**Published:** 2023-03-01

**Authors:** Andreas Samuelsson, David Bock, Mattias Prytz, Mattias Block, Carolina Ehrencrona, Anette Wedin, Eva Angenete, Eva Haglind

**Affiliations:** 1 Department of Surgery, SSORG – Scandinavian Surgical Outcomes Research Group, Institute of Clinical Sciences, Sahlgrenska Academy, University of Gothenburg, Gothenburg, Sweden; 2 Department of Surgery, Region Västra Götaland, NU-Hospital Group, Trollhättan, Sweden; 3 BioPharma Early Biometrics and Statistical Innovation, Data Science & AI, BioPharmaceuticals R&D, AstraZeneca, Gothenburg, Sweden; 4 Department of Research and Development, NU-Hospital Group, Trollhättan, Sweden; 5 Department of Surgery, Region Västra Götaland, Sahlgrenska University Hospital, Gothenburg, Sweden

## Abstract

**Background:**

Laparoscopic lavage as a treatment for perforated diverticulitis, Hinchey III, has been found safe and feasible in randomized trials. A few studies have reported functional outcomes and quality of life as secondary outcomes. This study investigated distress associated with dysfunction of the bowel or stoma, functional outcomes, and quality of life 2–3 years after surgery in a national unselected cohort.

**Methods:**

All patients in Sweden who underwent emergency surgery for perforated diverticulitis with purulent peritonitis (2016–2018) were invited to answer a comprehensive, study-specific questionnaire 2–3 years after the index surgery.

**Results:**

Out of 499 potential patients, 226 returned the questionnaire, and 209 were included in the analysis. There was no statistically significant difference between laparoscopic lavage and resection in distress associated with dysfunction of the bowel or stoma (odds ratio [OR], 1.32 [95% CI, 0.91–1.92]; *p* = 0.015). Bowel dysfunction measured by the LARS score was significantly higher for the lavage group (OR, 1.65 [95% CI, 1.11–2.45]), while stoma was more frequent after resection surgery (40 vs 6%).

**Conclusions:**

Patients experienced long-term distress from bodily dysfunction after emergency surgery for perforated diverticulitis regardless of the technique used. Regular follow-up could benefit these patients.

**Trial Registration:**

The project was registered at ClinicalTrials.gov on 2017–11-06. Identifier: NCT03332550. Acronym: LapLav.

**Supplementary Information:**

The online version contains supplementary material available at 10.1007/s00268-023-06961-2.

## Introduction

Laparoscopic lavage has been shown to be a feasible and safe treatment for perforated diverticulitis with purulent peritonitis in several randomized controlled studies, resulting in fewer patients in need of further surgery, shorter length of stays, and fewer patients with a stoma at 12 months compared with Hartmann’s procedure [1–3]. Health-economic analyses have suggested a cost–benefit advantage compared with resection surgery [4, 5]. Infectious complications were more commonly reported among patients that underwent laparoscopic lavage [6, 7]. Across studies with long-term follow-up [8–10], the reported rate of recurrent diverticulitis after laparoscopic lavage within 1 year was about 20%.

Emergency colorectal surgery to treat perforated diverticulitis with purulent peritonitis often entails postoperative functional disorders that can reduce quality of life (QoL). Two prior randomized trials, SCANDIV and DILALA, have included patient-reported functional outcome measures and QoL as secondary endpoints. In these trials, no significant differences in QoL or bodily functions were found between patients operated on by laparoscopic lavage or resection [2, 11]. However, considering that neither study had an adequate sample size nor statistical power for the evaluation of these endpoints, the need for further studies in larger, unselected cohorts is evident.

This study aimed to compare patient-reported functional outcomes and QoL 2–3 years after emergency laparoscopic lavage or resection for perforated diverticulitis with purulent peritonitis in a national cohort.

The hypothesis was that distress due to changes in bodily functions is less pronounced after laparoscopic lavage than after emergency resection when used a treatment for perforated diverticulitis, Hinchey III.

## Methods

### Study design

The study population was a national cohort of patients who received surgical treatment for perforated diverticulitis with purulent peritonitis, Hinchey III, during 2016–2018 in Sweden. The cohort was identified from the National Patient Register (Swedish Board of Health and Welfare) using ICD-10 and NOMESCO codes in combination with a code, indicating “emergency” care. Medical records for all patients were retrieved. In order to judge if a patient suffered from perforated diverticulitis, Hinchey III, the medical records from the index surgery were read using a set of predetermined points. When the surgery notes were found to be unclear on this point, the senior author read them separately and had the final decision. The study population and overall design have been described previously [12]. The report adheres to the STROBE statement.

### Patients and data collection

All patients alive 2–3 years after the index surgery were contacted by letter with the study information. Consent was retrieved by a research nurse over the telephone or by letter. A comprehensive, study-specific questionnaire with a prepaid return envelope was subsequently posted to each study participant.

Data retrieved from the National Patient Register and medical records were extracted into a prespecified Case Record Form. In cases when data between the Case Record Form and questionnaires differed, such as demographic information, the Case Record Form was used.

The questionnaire contained a total of 124 questions covering aspects including self-reported demographic and socioeconomic information, comorbidities, physical and mental well-being, and overall QoL. Additional questions examined the prevalence, severity, intensity, duration of bowel, sexual, and urinary dysfunction and associated distress for each function category. Questions on bodily function were constructed using a clinimetric approach [13]. Questions were selected from a question bank consisting of several hundred questions previously constructed by our group using in-depth interviews and content analysis in agreement with the clinimetric method [14].

The questionnaire first draft was validated by experts, ensuring its relevance to the study aims, and face-validated with patients to ascertain comprehensibility and relatability. In addition to the questionnaire, Audit-C [15], low anterior resection syndrome (LARS) [16], and EQ-5D-5L [17] instruments were administered.

### Aims

The primary aim was to compare distress associated with dysfunction of the bowel or stoma 2–3 years after emergency surgery due to perforated diverticulitis with purulent peritonitis. This aim reflects an intention-to-treat approach where the outcome of the intervention is assessed regardless of a potential postoperative formation or reversal of a colostomy or a colon resection.

The secondary aims were to characterize:

- Bowel function and distress
- Stoma function and distress
- Urinary function and distress
- Sexual function and distress
- QoL

as assessed by the specific questions/instrument in the questionnaire.

Distress due to bowel dysfunction was assessed by the question “How would you feel if this last month's bowel impairment were to remain the same for the rest of your life?” with response categories: *0* = *not applicable, I have no problems with bowel impairment, 1* = *it would not bother me at all, 2* = *it would bother me slightly, 3* = *it would bother me moderately, 4* = *it would bother me very much*.

For patients with a stoma, the corresponding question was: “*How would you feel if this last month’s stoma impairments were to remain the same for the rest of your life?*”, with the same five response categories as in the previous question.

The study’s primary endpoint, further surgery within one and two years, respectively, will be analyzed and presented in a separate report.

### Statistics

In the reporting of the primary outcome, there were a total of 140 and 265 evaluable patients in the laparoscopic lavage and resection groups, respectively [12]. Assuming a response rate of 75% for the questionnaire, 105 and 199 patients were projected to have evaluable data, respectively. For the question on distress associated with bowel or stoma dysfunction, it was assumed that the resection group had a distribution of 0.15, 0.2, 0.3, 0.2, and 0.15 for each of the five response categories. Based on these assumptions and using a two-sided test with a 5% significance level, the study provided 45% power to detect a reduction of 32.5% in the proportional odds of distress by lavage compared with resection. Thus, the study had 90% power to detect a 50% reduction. The sample size of the study was calculated for the primary endpoint “further surgery within one and two years.”

To make statistical comparisons between laparoscopic lavage and resection, we used an ordered logit regression model for ordinal and continuous outcomes [18, 19]. The causal model used the variables of age, cardiovascular disease, diabetes, chronic obstructive pulmonary disease, septicemia, and immunosuppressive therapy, which were previously identified as confounders with the type of surgery received for the same study cohort [12]. The propensity score calculated by Samuelsson et al. [12] was used for the inverse probability of treatment weighting [20]. The extent of balance between the two synthetic groups created by the weighting is shown in the Supplement. The derived weights were used in a weighted univariate regression model with the surgical procedure as a fixed effect. The main analysis was a multiple regression where the confounders were added as covariates to the weighted regression. R software [21] was used for the analysis. Estimation of the propensity score and weighting was performed using the twang package [22] and the ordinal package [23] for subsequent analyses. Results are presented as ORs and 95% CIs for adjusted (main) and unadjusted (supportive) analyses.

## Results

**Fig. 1:**
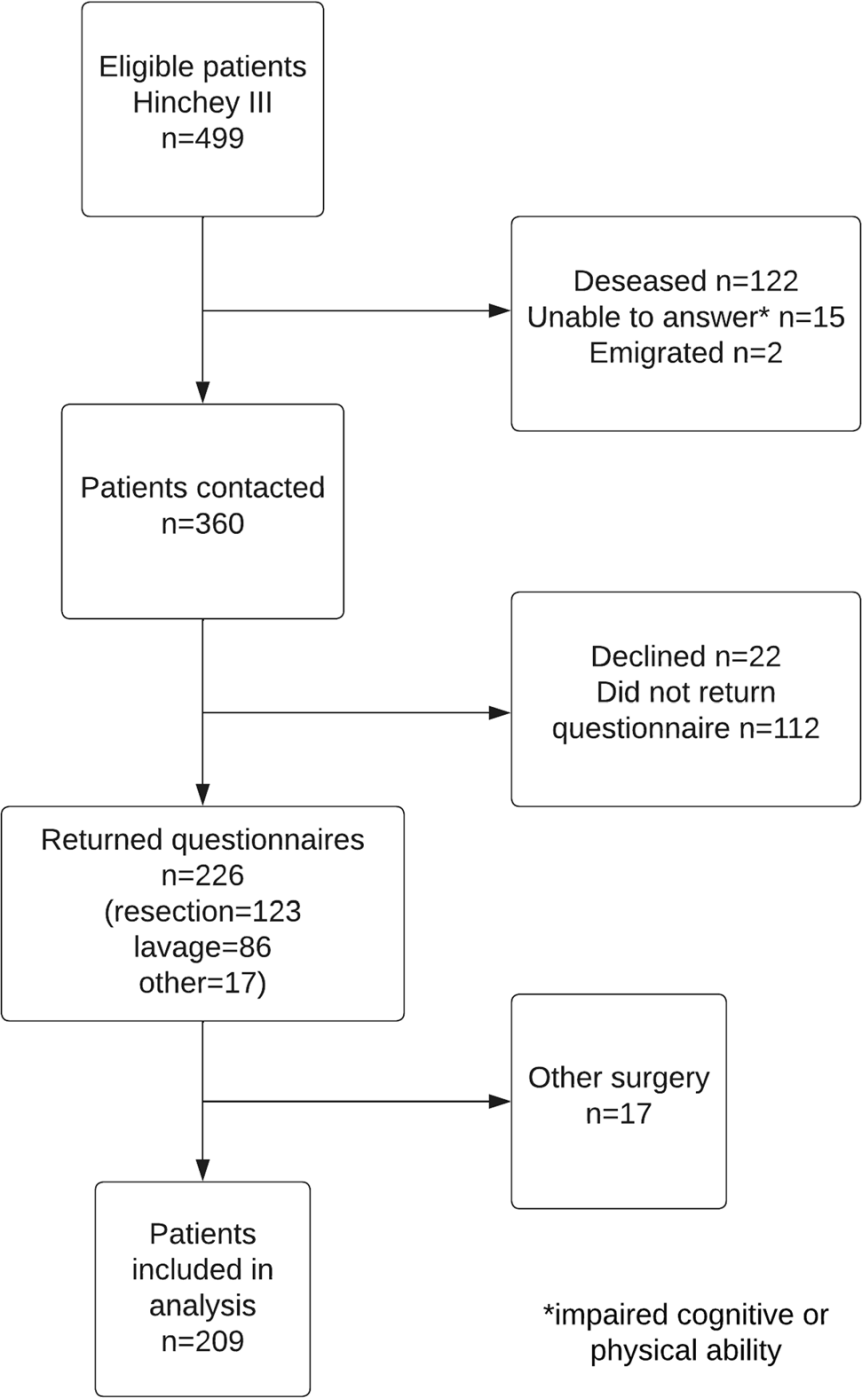
Flowchart

The study included 226 patients that had an emergency operation for perforated diverticulitis due to Hinchey grade III and returned the questionnaire 2–3 years after the index surgery (Fig. 1). In total, 209 patients were considered eligible for the current analysis; 123 had surgical resection, and 86 had laparoscopic lavage. The remaining 17 patients were excluded based on the type of index surgery.

Patient characteristics derived from medical records are summarized in Table 1. The resection group was older and had more comorbidities. Patient-reported characteristics from the questionnaires are summarized in Table 2. More patients in the resection group had cortisone treatment or rheumatoid arthritis, while university education was almost twice as common in the lavage group. As would be expected, patients in the resection group had a higher stoma frequency (49/123 vs 5/86).

**Table 1:** Clinical demography

Characteristic	Overall *N* = 464	Resection *N* = 123	Lavage *N* = 86	Resection (non-responder) *N* = 168	Lavage (non-responder) *N* = 87
Age, median (IQR)	68 (56, 77)	68 (59, 75)	62 (50, 68)	72 (65, 80)	66 (52, 80)
*Sex, n/N (%)*					
Female	243/464 (52%)	62/123 (50%)	41/86 (48%)	99/168 (59%)	41/87 (47%)
Male	221/464 (48%)	61/123 (50%)	45/86 (52%)	69/168 (41%)	46/87 (53%)
*Diabetes, n/N (%)*					
No	419/464 (90%)	108/123 (88%)	82/86 (95%)	148/168 (88%)	81/87 (93%)
Yes	41/464 (8.8%)	13/123 (11%)	4/86 (4.7%)	18/168 (11%)	6/87 (6.9%)
Don’t know	4/464 (0.9%)	2/123 (1.6%)	0/86 (0%)	2/168 (1.2%)	0/87 (0%)
*Comorbidity, n/N (%)*					
No	193/464 (42%)	43/123 (35%)	54/86 (63%)	52/168 (31%)	44/87 (51%)
Yes	266/464 (57%)	78/123 (63%)	32/86 (37%)	113/168 (67%)	43/87 (49%)
Don’t know	5/464 (1.1%)	2/123 (1.6%)	0/86 (0%)	3/168 (1.8%)	0/87 (0%)
*COPD, n/N (%)*					
No	424/464 (91%)	118/123 (96%)	86/86 (100%)	141/168 (84%)	79/87 (91%)
Yes	40/464 (8.6%)	5/123 (4.1%)	0/86 (0%)	27/168 (16%)	8/87 (9.2%)
*Cardiovascular disease, n/N (%)*					
No	218/464 (47%)	47/123 (38%)	56/86 (65%)	66/168 (39%)	49/87 (56%)
Yes	246/464 (53%)	76/123 (62%)	30/86 (35%)	102/168 (61%)	38/87 (44%)
*Immunosuppressed, n/N (%)*					
No	332/464 (72%)	89/123 (72%)	80/86 (93%)	92/168 (55%)	71/87 (82%)
Yes	132/464 (28%)	34/123 (28%)	6/86 (7.0%)	76/168 (45%)	16/87 (18%)
*Sepsis at surgery, n/N (%)*					
No	381/464 (82%)	106/123 (86%)	80/86 (93%)	118/168 (70%)	77/87 (89%)
Yes	83/464 (18%)	17/123 (14%)	6/86 (7.0%)	50/168 (30%)	10/87 (11%)

**Table 2:** Patient-reported demography

Characteristic	Overall *N* = 209	Resection *N* = 123	Lavage *N* = 86
Smoking status, n/N (%) *			
Non-smoker	86/209 (41%)	48/123 (39%)	38/86 (44%)
Former smoker	98/209 (47%)	61/123 (50%)	37/86 (43%)
Current smoker	25/209 (12%)	14/123 (11%)	11/86 (13%)
AUDIT-C score, median (IQR)	2.00 (1.00, 4.00)	2.00 (0.00, 4.00)	3.00 (1.00, 4.00)
Unknown	10	8	2
Body mass index (BMI), median (IQR)	26.5 (23.7, 30.2)	26.6 (23.8, 30.6)	26.1 (23.7, 30.1)
Unknown	3	2	1
Physical activity, n/N (%) **			
Physically inactive	40/209 (19%)	29/123 (24%)	11/86 (13%)
Some light physical activity	143/209 (68%)	81/123 (66%)	62/86 (72%)
Regular physical activity and training	24/209 (11%)	13/123 (11%)	11/86 (13%)
Regular hard physical training for competition sports	2/209 (1.0%)	0/123 (0%)	2/86 (2.3%)
*Cortisone treatment, n/N (%)*			
Yes	39/200 (20%)	32/117 (27%)	7/83 (8.4%)
No	159/200 (80%)	83/117 (71%)	76/83 (92%)
Don’t know	2/200 (1.0%)	2/117 (1.7%)	0/83 (0%)
Unknown	9	6	3
*Rheumatoid arthritis, n/N (%)*			
Yes	15/206 (7.3%)	12/120 (10%)	3/86 (3.5%)
No	187/206 (91%)	107/120 (89%)	80/86 (93%)
Don’t know	4/206 (1.9%)	1/120 (0.8%)	3/86 (3.5%)
Unknown	3	3	0
*Crohn’s disease or ulcerative colitis, n/N (%)*			
Yes	2/208 (1.0%)	0/122 (0%)	2/86 (2.3%)
No	205/208 (99%)	121/122 (99%)	84/86 (98%)
Don’t know	1/208 (0.5%)	1/122 (0.8%)	0/86 (0%)
Unknown	1	1	0
*Residence, n/N (%)*			
Rural area	36/206 (17%)	21/120 (18%)	15/86 (17%)
Small/middle-size town	138/206 (67%)	82/120 (68%)	56/86 (65%)
Bigger city	32/206 (16%)	17/120 (14%)	15/86 (17%)
Unknown	3	3	0
*Education, n/N (%)*			
No university education	161/207 (78%)	101/121 (83%)	60/86 (70%)
University education	46/207 (22%)	20/121 (17%)	26/86 (30%)
Unknown	2	2	0
*Stoma, n/N (%)*			
Stoma: no	155/209 (74%)	74/123 (60%)	81/86 (94%)
Stoma: yes	54/209 (26%)	49/123 (40%)	5/86 (5.8%)
*Marital status, n/N (%)*			
Not in a relationship	53/208 (25%)	40/122 (33%)	13/86 (15%)
In a relationship	155/208 (75%)	82/122 (67%)	73/86 (85%)
Unknown	1	1	0
*Employment status, n/N (%)*			
Retired/unemployed	136/207 (66%)	91/121 (75%)	45/86 (52%)
Employed	71/207 (34%)	30/121 (25%)	41/86 (48%)
Unknown	2	2	0

^*^According to definitions used by the Centers for Disease Control and Prevention, CDC

^**^Saltin–Grimby Physical Activity Level Scale

**Fig. 2:**
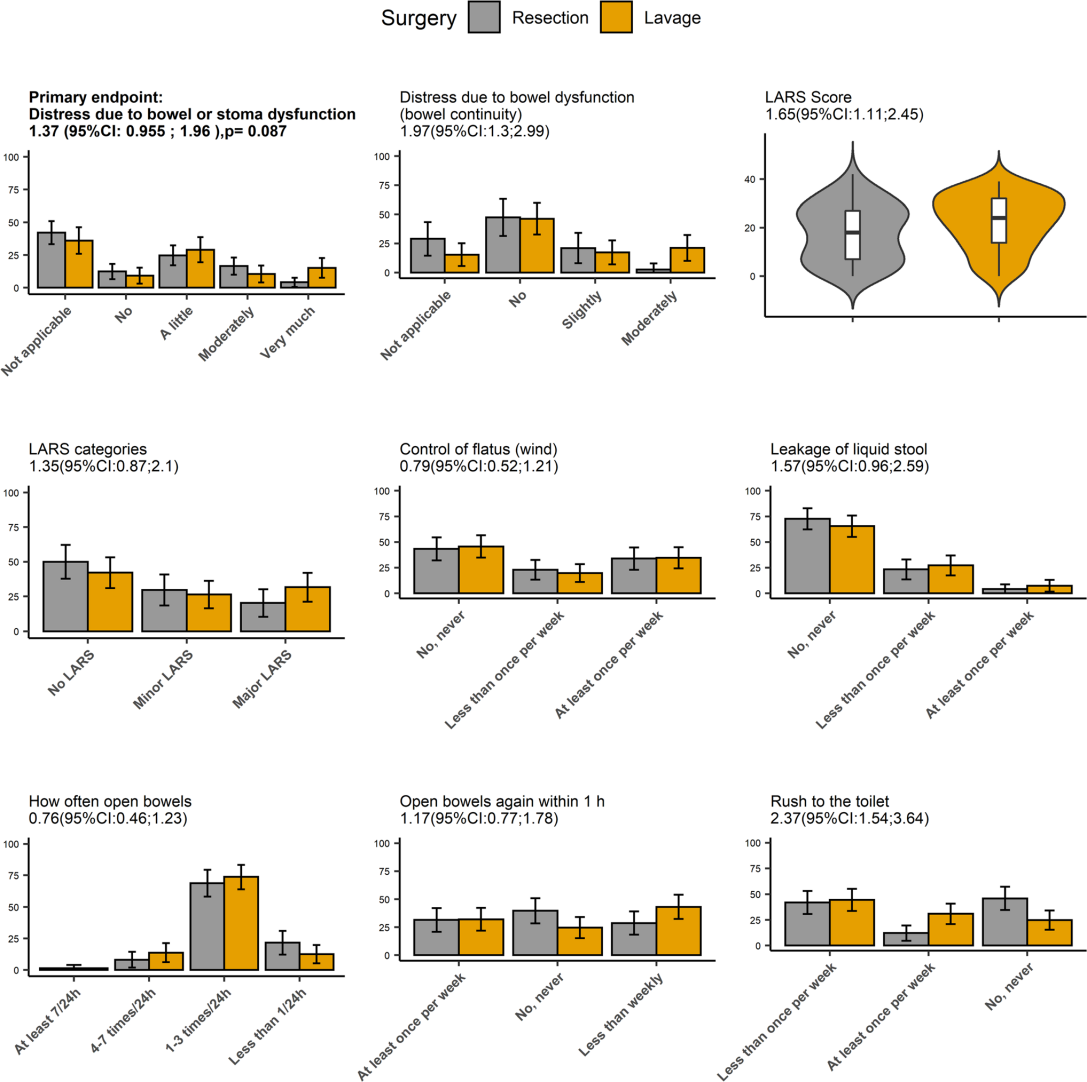
Bowel function

The study’s primary outcome, comparison of distress from bowel dysfunction regardless of bowel continuity or stoma, did not differ significantly between laparoscopic lavage and resection (OR, 1.32 [95% CI, 0.91–1.92]; p = 0.15 adjusted analysis) (Fig. 2). In those with bowel continuity, however, the lavage group had significantly higher distress due to bowel dysfunction (OR, 1.98 [95% CI, 1.29; 3.06]; *p* = 0.002) and a significantly higher LARS score compared with the resection group (OR, 1.65 [95% CI, 1.11–2.45]). When the five questions of the LARS instrument were analyzed separately, “having to rush to the toilet” differed significantly in favor of resection (OR, 2.37 [95% CI, 1.54–3.64]).

**Fig. 3:**
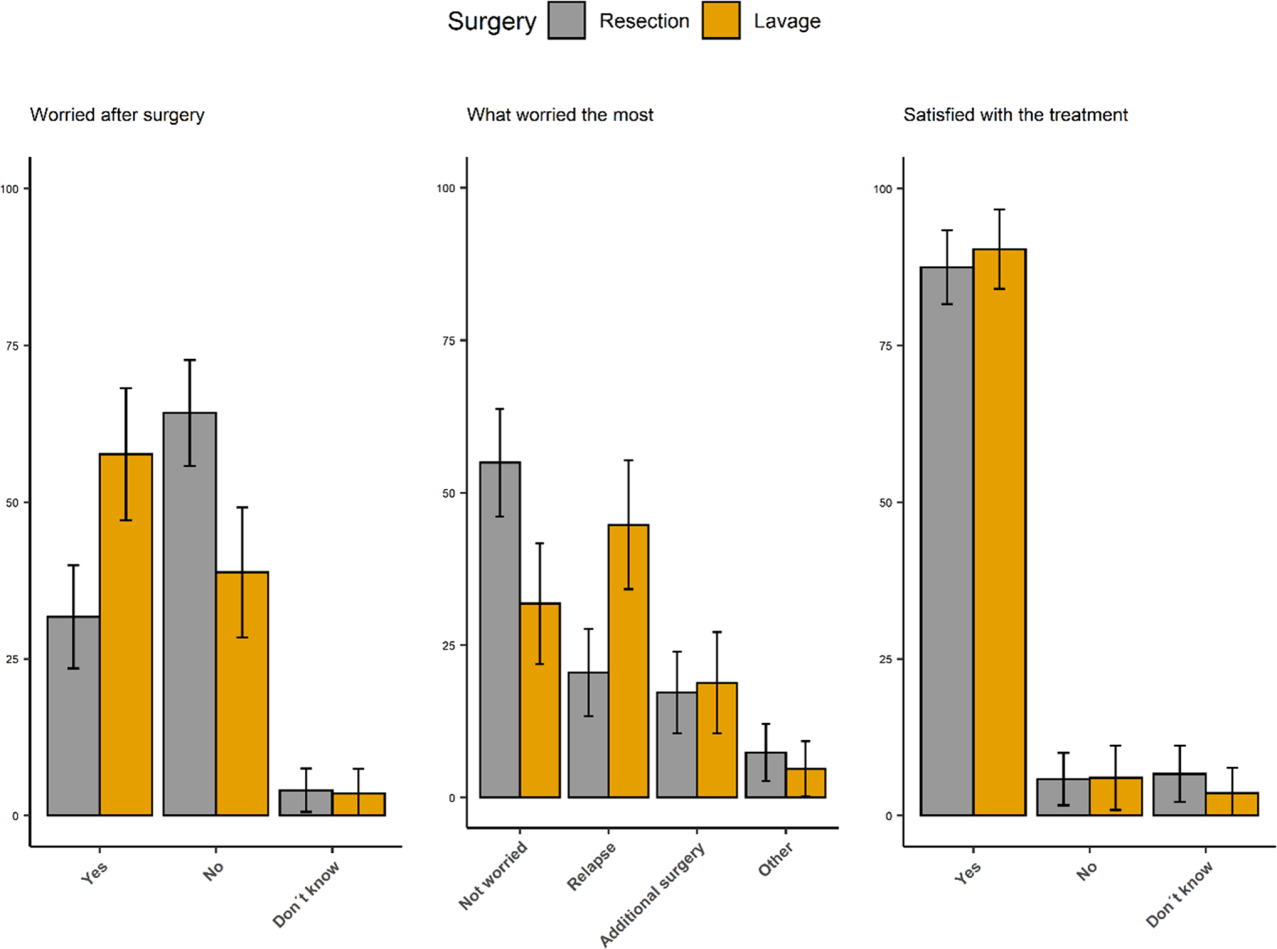
Worries after surgery

About one-third of the patients with a stoma reported moderate-to-high overall distress from their stoma. Only 20% recalled being involved in the decision to perform an operation resulting in a stoma (Table 3). Patients operated with lavage worried more about renewed perforated diverticulitis compared with those in the resection group. However, satisfaction with treatment 2–3 years following surgery did not differ between the two groups (Fig. 3).

**Table 3:** Stoma function

	Resection	Lavage
*Type of stoma*		
Ileostomy	5(10%)	0
Colostomy	31(65%)	4(80%)
Don’t know	12(25%)	1(20%)
*When did you get a stoma?*		
Had it before perforated diverticulitis	2(4.3%)	NA
During diagnosis and treatment of perforated diverticulitis	44(96%)	NA
After acute treatment of perforated diverticulitis	0	3(100%)
*Feel involved in the decision to have a stoma?*		
No	29(62%)	1(20%)
Yes	8(17%)	3(60%)
Don’t know/can’t remember	10(21%)	1(20%)
*Had loud gas departures from the stoma, last month?*		
No	8(16%)	1(25%)
At least 1/month	7(14%)	NA
At least 1/week	7(14%)	NA
At least 3/week	9(18%)	1(25%)
At least daily	18(37%)	2(50%)
*Distress due to loud gas departures?*		
No	14(29%)	2(40%)
N/A	7(15%)	NA
A little	11(23%)	1(20%)
Moderately	11(23%)	1(20%)
Very much	5(10%)	1(20%)
*Had foul-smelling gas escapes from the stoma, last month?*		
No	24(50%)	4(80%)
At least 1/month	10(21%)	NA
At least 1/week	4(8.3%)	NA
At least 3/week	6(12%)	NA
At least daily	4(8.3%)	1(20%)
*Distress due to foul-smelling gas escapes?*		
No	10(21%)	1(20%)
N/A	22(46%)	2(40%)
A little	5(10%)	NA
Moderately	4(8.3%)	NA
Very much	7(15%)	2(40%)
*Had a leak of stool from the stoma, last month?*		
No	26(54%)	1(20%)
At least 1/month	15(31%)	2(40%)
At least 1/week	6(12%)	2(40%)
At least daily	1(2.1%)	NA
*Distress due to leak of stool?*		
No	3(6.2%)	NA
N/A	25(52%)	1(20%)
A little	5(10%)	2(40%)
Moderately	4(8.3%)	NA
Very much	11(23%)	2(40%)
*Worried about the stoma leaking, last month?*		
N/A. Not worried	24(50%)	1(20%)
At least 1/month	8(17%)	3(60%)
At least 1/week	6(12%)	NA
At least 3/week	2(4.2%)	NA
At least daily	8(17%)	1(20%)
*Skin around the stoma been irritated, last month?*		
No	20(42%)	1(20%)
A little	18(38%)	2(40%)
Moderately	7(15%)	1(20%)
Very much	3(6.2%)	1(20%)
*Problems taking care of your stoma, last month?*		
No	41(87%)	4(80%)
A little	3(6.4%)	1(20%)
Moderately	2(4.3%)	NA
Very much	1(2.1%)	NA
*Further surgery due to problems with the stoma?*		
No	44(90%)	3(60%)
Yes	5(10%)	2(40%)
*You or a doctor noticed a bulge (hernia) at the stoma?*		
Don’t know	9(18%)	1(20%)
No	14(29%)	3(60%)
Yes	26(53%)	1(20%)
*Distress due to overall problems with the stoma?*		
No	4(8.3%)	NA
N/A	16(33%)	2(40%)
A little	12(25%)	1(20%)
Moderately	12(25%)	NA
Very much	4(8.3%)	2(40%)
*I can live a full life with my stoma*		
Not true	8(16%)	NA
Partly true	13(27%)	3(60%)
Largely true	19(39%)	2(40%)
Fully true	9(18%)	NA
*I feel comfortable with my stoma*		
Not true	7(14%)	NA
Partly true	14(29%)	3(60%)
Largely true	16(33%)	2(40%)
Fully true	12(24%)	NA
*Worried something embarrassing can happen during sexual activity due to my stoma?*		
Not true	7(58%)	2(50%)
Partly true	2(17%)	NA
Largely true	1(8.3%)	NA
Fully true	2(17%)	2(50%)
*I feel dirty and unclean due to my stoma*		
Not true	25(51%)	3(60%)
Partly true	12(24%)	2(40%)
Largely true	5(10%)	NA
Fully true	7(14%)	NA
*I have the leisure activities and social life I desire*		
Not true	10(20%)	1(20%)
Partly true	17(35%)	2(40%)
Largely true	13(27%)	1(20%)
Fully true	9(18%)	1(20%)

**Fig. 4:**
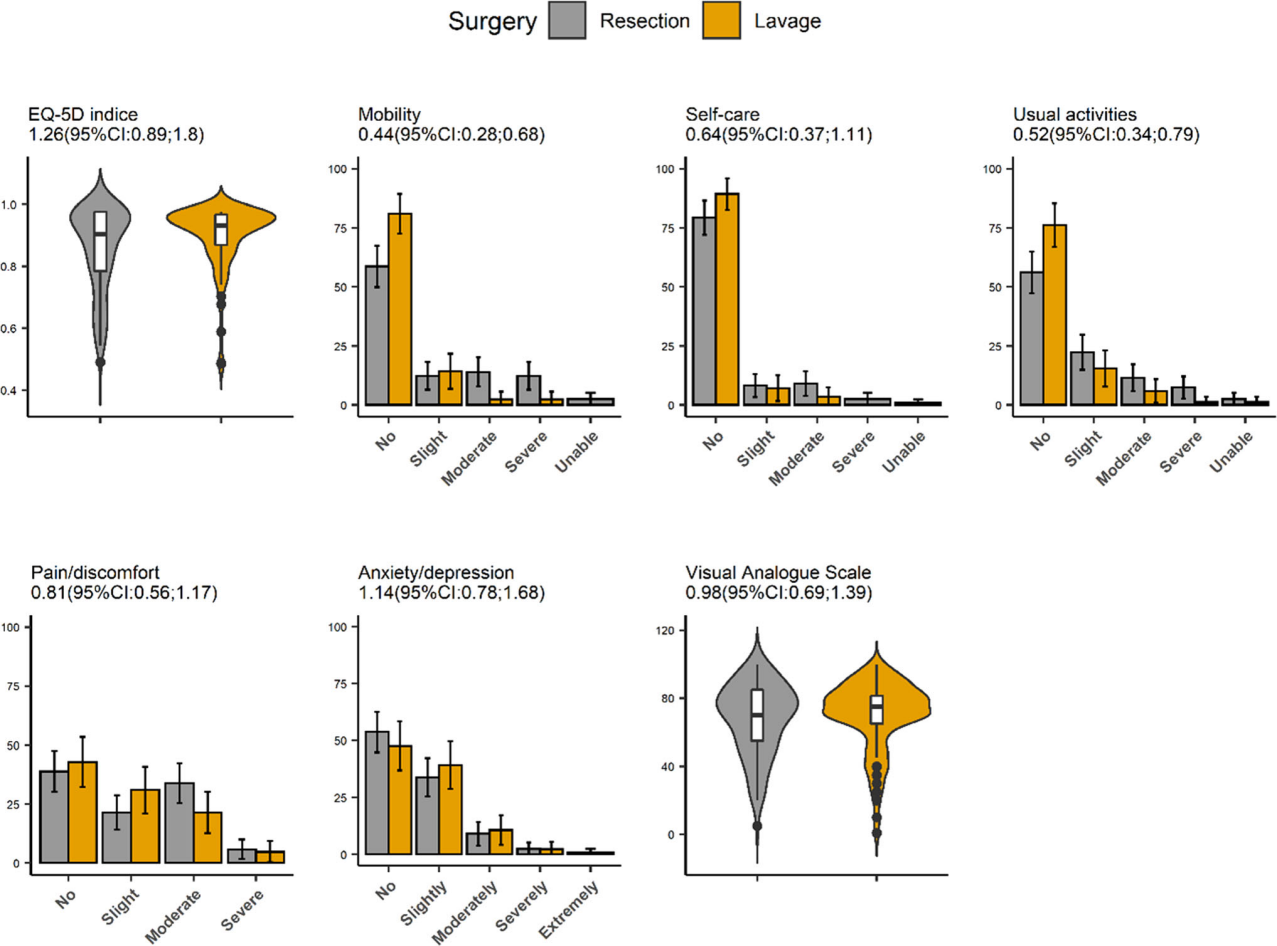
QoL

Patient’s reported further surgery due to recurrent problems in 4/123 cases in the resection group and in 7/86 cases in the lavage group (3% vs 8%). Urinary function was similar between the two groups (Fig. A, Supplement), while sexual activity and satisfaction with sexual health numerically favored the lavage group (Fig. B, Supplement). No difference in overall health-related QoL, measured by the EQ-5D-5L [17], was found. Scores in the dimensions of mobility, self-care, and usual activities were better in the lavage group, but these differences were not statistically significant (Fig. 4).

## Discussion

This study leveraged the Swedish national registry to examine patient-reported functional outcomes, distress, and QoL in patients who had surgical treatment for perforated diverticulitis with purulent peritonitis between 2016 and 2018. We used a combination of variables to examine dysfunction of the intact bowel and stoma in those with bowel continuity and stoma, respectively. This was intended to avoid bias resulting from the unequal distribution of permanent stoma between the two groups. We found that, at 2–3 years post-index surgery, there was no difference in reported overall distress related to bowel or stoma dysfunction between patients who had laparoscopic lavage and those who had resection. Yet, among patients with bowel continuity, distress associated with bowel dysfunction was significantly higher in the lavage group. This finding could be attributed to the impairment of bowel function as reflected by the higher LARS score and underpinned by a more frequent need to rush to the toilet.

It is perhaps unsurprising that patients with a remaining sigmoid colon after perforated diverticulitis had a high LARS score. A severe episode of diverticulitis most probably leads to fibrosis and a less compliant rectosigmoid, which in turn could increase the risk for urgency. It is interesting to compare the LARS scores in the current study cohort with those reported for the general population from several countries [24, 25]. From such reports, it appears that 10–15% of the general population report major LARS; thus, it is hard to truly evaluate the functional outcome in these patients without a baseline measurement [26]. We refrained from asking about baseline function in the questionnaire, as it was completed 2–3 years after the emergency operation and recall bias would be impossible to control.

Among the study population, permanent stoma (2–3 years after emergency surgery) was common after emergency resection. This is consistent with the fact that Hartmann’s procedure was the dominant resection procedure applied in emergency surgery for perforated diverticulitis in Sweden during the study period. Few patients (6%) in the lavage group had a stoma because of a reoperation; consequently, statistical comparisons between groups regarding stoma function were not considered meaningful. Only a few patients recalled being involved in the decision to create a stoma, which might be expected due to the emergency nature of surgery as well as recall bias. This could theoretically influence patients’ acceptance of their stoma and their coping ability, but the cohort, albeit large, was not of sufficient size to analyze such associations. Indeed, in comparison with patients who underwent elective abdominoperineal surgery for rectal cancer in a national cohort in Sweden, patients in this study of diverticulitis reported less acceptance of their stoma [27]. It should be emphasized that the majority of patients with an elective stoma after rectal cancer surgery accept their situation and live with no or few constraints [28].

In the current study cohort, we found that patients in the lavage group worried more about recurrent disease. This seems reasonable given the previously reported recurrence rates of about 20% after lavage [8] and that this risk may have been communicated to patients pre- or postoperatively. It is feasible that a combination of symptoms such as urgency, as evidenced by the LARS assessment, and associated fear of a new emergency procedure may contribute to increased worry for relapse after lavage, whereas dysfunction and distress about stoma, as well as fear of the next procedure, could influence distress after Hartman’s procedure. Ultimately, however, there was no difference in the proportion of patients with further surgery between the two groups in this cohort.

Urinary and sexual functions did not differ noticeably between the two groups, which was not surprising as neither surgical technique is expected to damage pelvic nerves. Findings regarding sexual activity and general satisfaction with sexual health should not be regarded as clinically significant as residual confounding cannot be disregarded. Additionally, we found that QoL using the EQ-5D-5L was comparable regardless of surgical approach. This is in line with previous findings by Andersson et al. who reported no differences in QoL measured using validated instruments between laparoscopic and open surgery for rectal cancer in the long term [29]. It has been suggested that in patients with recurrent diverticulitis, QoL improves after resection [30, 31]. It is possible that the measuring point 2–3 years after surgery may not be the ideal point to compare laparoscopic and open surgery in general, but regarding late functional outcomes, it is reasonable follow-up of patients after an emergency operation for perforated diverticulitis, regardless of surgical technique used, should include evaluation of both function and distress.

Perforated diverticulitis with purulent peritonitis is a rare condition, and the national and relatively large cohort is a strength of this study. We used an intention-to-treat policy when analyzing the groups which should be relevant both from a patient perspective and also for the surgeon when choosing technique in practice.

The non-randomized assignment to treatment groups is a limitation from a causality assessment perspective. We limited the influence of confounders in the analyses, but additional unobserved confounding cannot be ruled out. The overall sample size was limited by the fact that 139 patients in the parent study were either deceased or cognitively impaired and could not be included in the current analysis (see Fig. 1). This can be seen as a limitation but should not represent a selection bias, as we have no indication that death or cognitive difficulties were more frequent in one of the groups. Of all included patients, 63% returned the questionnaire. The cohort size did not allow for subgroup analyses, and the group size was smaller than the post hoc power calculation. Further surgery could influence patient-reported outcome measures 2–3 years after index surgery. However, only eight percent in the lavage group reported having had additional surgery done due to recurrent problems. The study-specific questionnaire might present difficulties if later comparisons with results from other studies using different questions were to be made. Further, the LARS instrument and score have not been validated for patients with perforated diverticulitis. It has been used for other diagnoses than rectal cancer, for which it is validated for [26, 32].

## Conclusion

In the long term, patients treated by emergency surgery for perforated diverticulitis with purulent peritonitis continue to experience distress from bowel dysfunction regardless of bowel continuity or permanent stoma. We suggest that patients who have undergone emergency surgery due to perforated diverticulitis should be offered regular follow-ups regardless of the surgical technique used to diagnose and offer treatment for functional disorders.

## Supplementary Information

Below is the link to the electronic supplementary material.

Supplementary file1 (DOCX 35 kb)

Supplementary file2 (DOCX 1416 kb)

## Data Availability

The R code for data management and analysis is available at: https://github.com/dvdsb/Functional-outcomes-of-emergency-surgery-for-diverticulitis-Hinchey-III. Individual patient-level data cannot be made publicly available or shared due to restrictions in the ethics approval.

## References

[1] Vennix S, Musters GD, Mulder IM (2015). Laparoscopic peritoneal lavage or sigmoidectomy for perforated diverticulitis with purulent peritonitis: a multicentre, parallel-group, randomised, open-label trial. Lancet (London, England).

[2] Thornell A, Angenete E, Bisgaard T (2016). Laparoscopic lavage for perforated diverticulitis with purulent peritonitis: a randomized trial. Ann Intern Med.

[3] Schultz JK, Yaqub S, Wallon C (2015). Laparoscopic lavage vs primary resection for acute perforated diverticulitis: the SCANDIV randomized clinical trial. JAMA.

[4] Gehrman J, Angenete E, Bjorholt I (2016). Health economic analysis of laparoscopic lavage versus Hartmann's procedure for diverticulitis in the randomized DILALA trial. Br J Surg.

[5] Vennix S, van Dieren S, Opmeer BC (2017). Cost analysis of laparoscopic lavage compared with sigmoid resection for perforated diverticulitis in the Ladies trial. Br J Surg.

[6] Angenete E, Bock D, Rosenberg J (2017). Laparoscopic lavage is superior to colon resection for perforated purulent diverticulitis-a meta-analysis. Int J Colorectal Dis.

[7] Penna M, Markar SR, Mackenzie H et al (2017) Laparoscopic Lavage Versus Primary Resection for Acute Perforated Diverticulitis: Review and Meta-analysis. Ann Surg 10.1097/SLA.0000000000002236 28338510

[8] Azhar N, Johanssen A, Sundström T (2021). Laparoscopic lavage vs primary resection for acute perforated diverticulitis: long-term outcomes from the scandinavian diverticulitis (SCANDIV) randomized clinical trial. JAMA Surg.

[9] Kohl A, Rosenberg J, Bock D (2018). Two-year results of the randomized clinical trial DILALA comparing laparoscopic lavage with resection as treatment for perforated diverticulitis. Br J Surg.

[10] Sneiders D, Lambrichts DPV, Swank HA (2019). Long-term follow-up of a multicentre cohort study on laparoscopic peritoneal lavage for perforated diverticulitis. Colorectal Dis.

[11] Schultz JK, Wallon C, Blecic L (2017). One-year results of the SCANDIV randomized clinical trial of laparoscopic lavage versus primary resection for acute perforated diverticulitis. Br J Surg.

[12] Samuelsson A, Bock D, Prytz M (2021). Laparoscopic lavage for perforated diverticulitis in the LapLav study: population-based registry study. Br J Surg.

[13] Steineck G, Bergmark K, Henningsohn L (2002). Symptom documentation in cancer survivors as a basis for therapy modifications. Acta oncologica (Stockholm, Sweden).

[14] Thorsteinsdottir T, Stranne J, Carlsson S (2011). LAPPRO: a prospective multicentre comparative study of robot-assisted laparoscopic and retropubic radical prostatectomy for prostate cancer. Scand J Urol Nephrol.

[15] Bush K, Kivlahan DR, McDonell MB (1998). The AUDIT alcohol consumption questions (AUDIT-C): an effective brief screening test for problem drinking. Ambulatory Care Quality Improvement Project (ACQUIP). alcohol use disorders identification test. Arch Intern Med.

[16] Emmertsen KJ, Laurberg S (2012). Low anterior resection syndrome score: development and validation of a symptom-based scoring system for bowel dysfunction after low anterior resection for rectal cancer. Ann Surg.

[17] The EuroQol (1990) EuroQol—a new facility for the measurement of health-related quality of life. Health policy (Amsterdam, Netherlands) 16:199–208 10.1016/0168-8510(90)90421-9 10109801

[18] McCullagh P (1980). Regression models for ordinal data. J R Stat Soc Ser B Methodol.

[19] Liu Q, Shepherd BE, Li C (2017). Modeling continuous response variables using ordinal regression. Stat Med.

[20] Austin PC, Stuart EA (2015). Moving towards best practice when using inverse probability of treatment weighting (IPTW) using the propensity score to estimate causal treatment effects in observational studies. Stat Med.

[21] R Core Team (2020 R: A language and environment for statistical computing., R Foundation for Statistical Computing, Vienna, Austria. URL: https://www.R-project.org/.

[22] Cefalu M RG, McCaffrey D, Morral A, Griffin BA, Burgette L (2021) Toolkit for Weighting and Analysis of Nonequivalent Groups

[23] Christensen RHB ordinal—Regression Models for Ordinal Data (2019)

[24] Sandberg S, Asplund D, Bisgaard T (2020). Low anterior resection syndrome in a Scandinavian population of patients with rectal cancer: a longitudinal follow-up within the QoLiRECT study. Colorectal Dis.

[25] van Heinsbergen M, Van der Heijden JAG, Stassen LP (2020). The low anterior resection syndrome in a reference population: prevalence and predictive factors in the Netherlands. Colorectal Dis.

[26] Bock D, Angenete E, Gonzales E (2018). Assessing health, quality of life and urogenital function in a sample of the Swedish general population: a cross-sectional study. BMJ Open.

[27] Marinez AC, González E, Holm K (2016). Stoma-related symptoms in patients operated for rectal cancer with abdominoperineal excision. Int J Colorectal Dis.

[28] González E, Holm K, Wennström B (2016). Self-reported wellbeing and body image after abdominoperineal excision for rectal cancer. Int J Colorectal Dis.

[29] Andersson J, Angenete E, Gellerstedt M (2013). Health-related quality of life after laparoscopic and open surgery for rectal cancer in a randomized trial. Br J Surg.

[30] van de Wall BJM, Stam MAW, Draaisma WA (2017). Surgery versus conservative management for recurrent and ongoing left-sided diverticulitis (DIRECT trial): an open-label, multicentre, randomised controlled trial. Lancet Gastroenterol Hepatol.

[31] Santos A, Mentula P, Pinta T (2021). Comparing laparoscopic elective sigmoid resection with conservative treatment in improving quality of life of patients with diverticulitis: the laparoscopic elective sigmoid resection following diverticulitis (LASER) randomized clinical trial. JAMA Surg.

[32] Juul T, Elfeki H, Christensen P (2019). Normative data for the low anterior resection syndrome score (LARS Score). Ann Surg.

