# Supplementary material for: Functional Outcomes of Emergency Surgery for Perforated Diverticulitis, Hinchey Grade III
Source: World J Surg. 2023 Mar 1;47(6):1570–82. doi: 10.1007/s00268-023-06961-2 (PMC10156766; doi:10.1007/s00268-023-06961-2)
Supplement: Supplementary file 2 — Supplementary file2 (DOCX 1416 kb) [file 268_2023_6961_MOESM2_ESM.docx]

# Supplement:

# Urine function


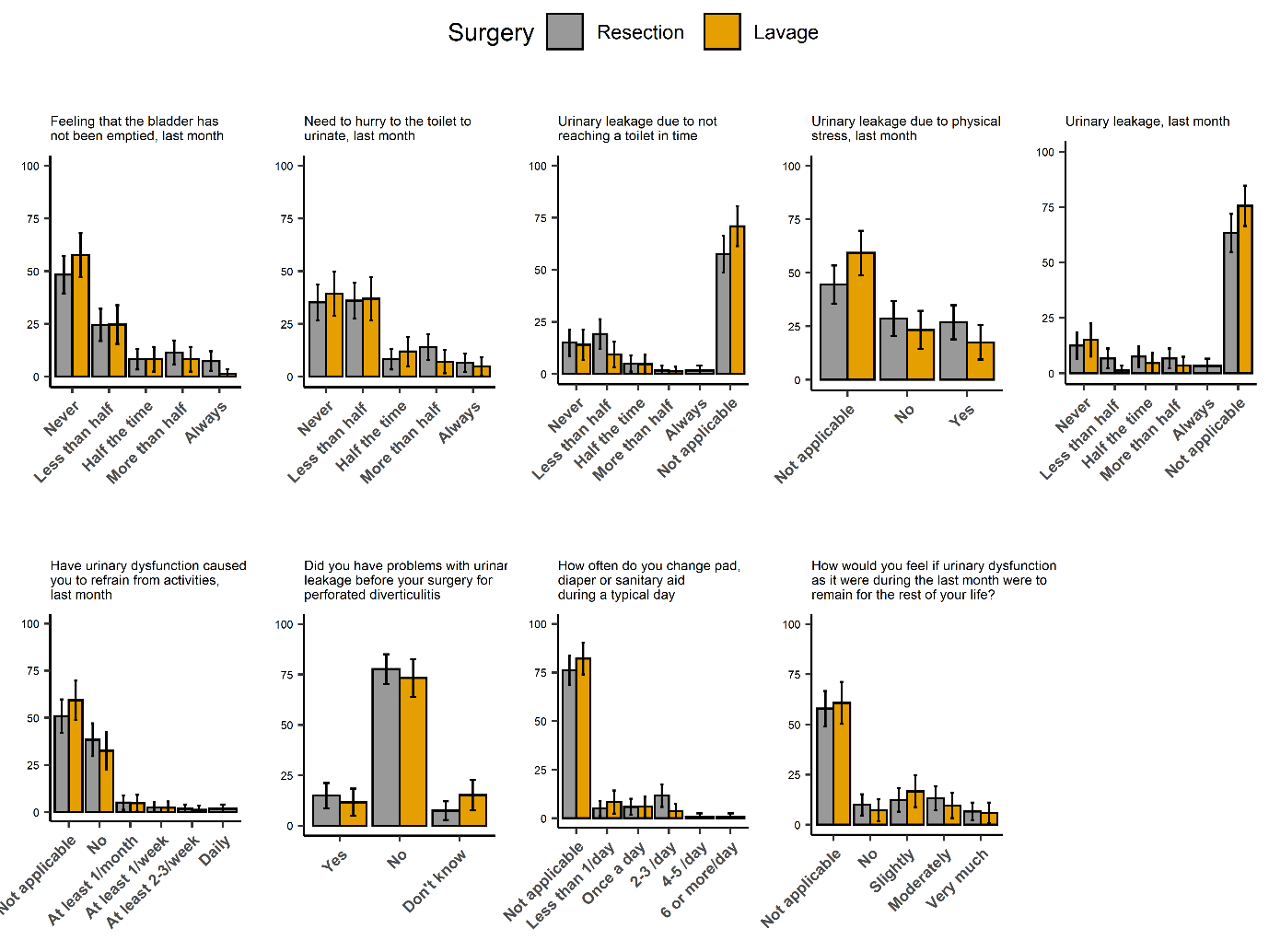


# Sex general


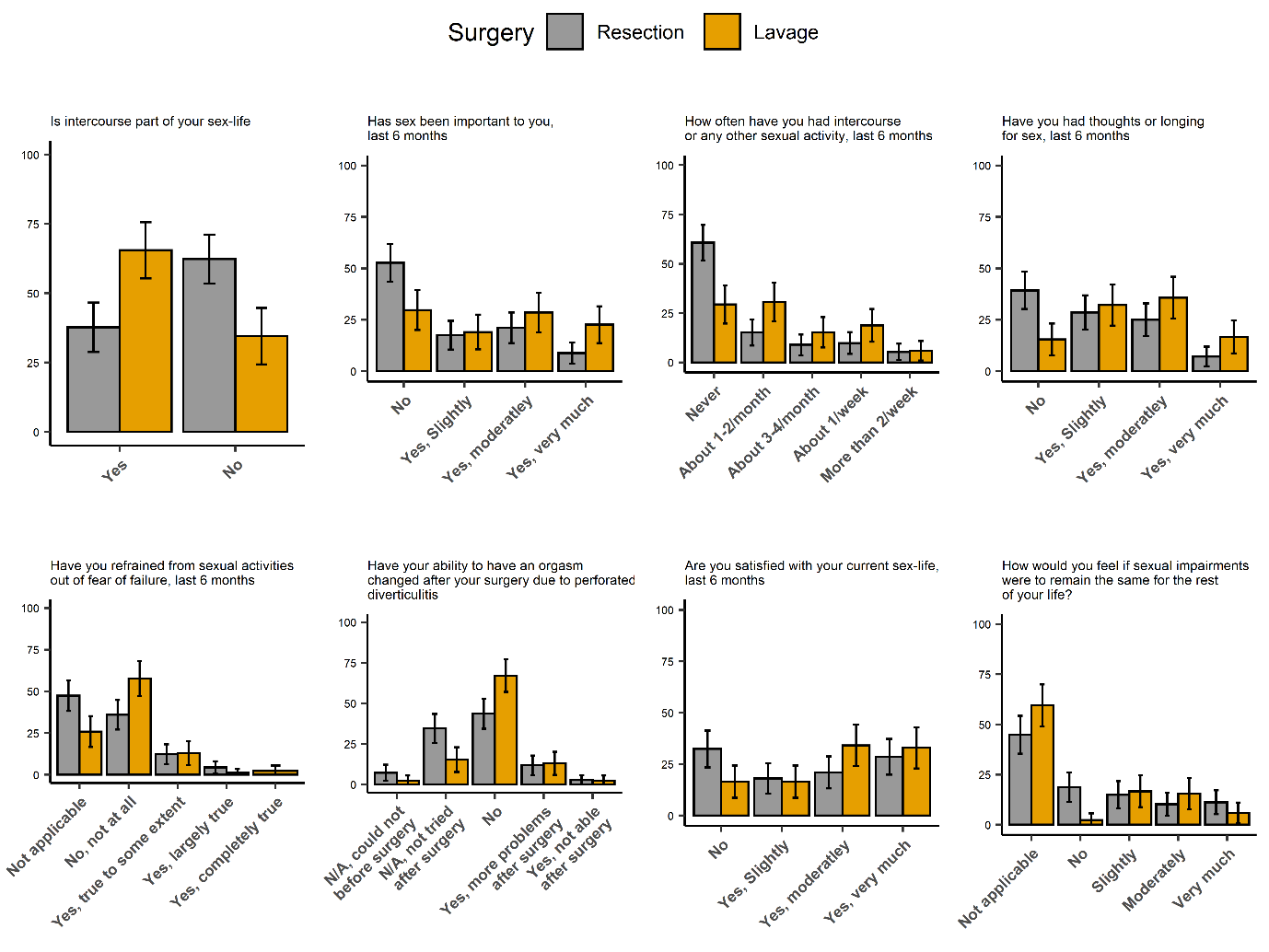


# Sex males


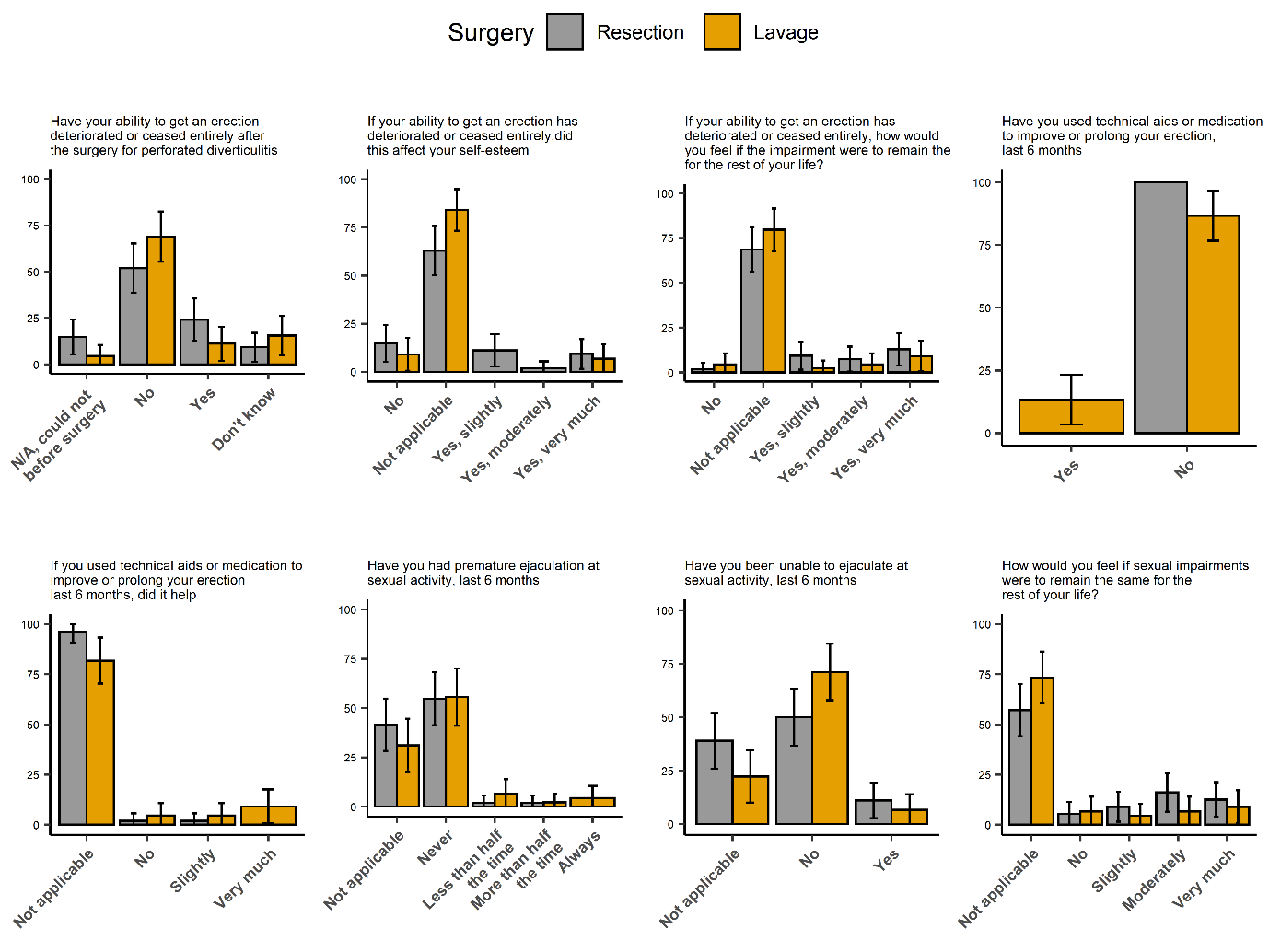


# Sex females


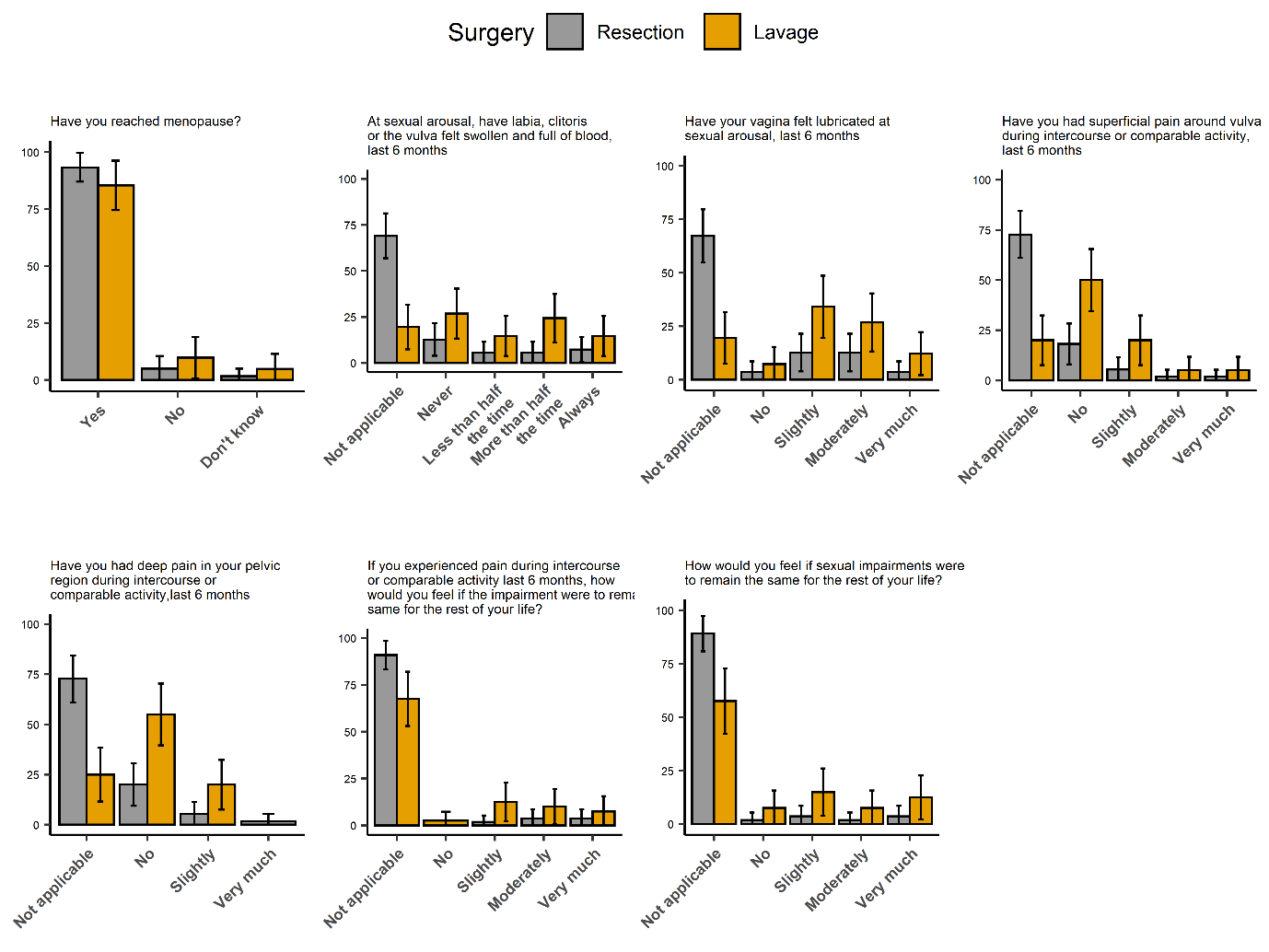


# Evaluation of the propensity score weights

The evaluation of the weights based on propensity scores that was done in Samuelsson (2021) is presented below. Generalized boosted regression with minimal average standardized effect size as optimality criteria was used to estimate the propensity score using the twang package. The average treatment effect was used for deriving the weights.


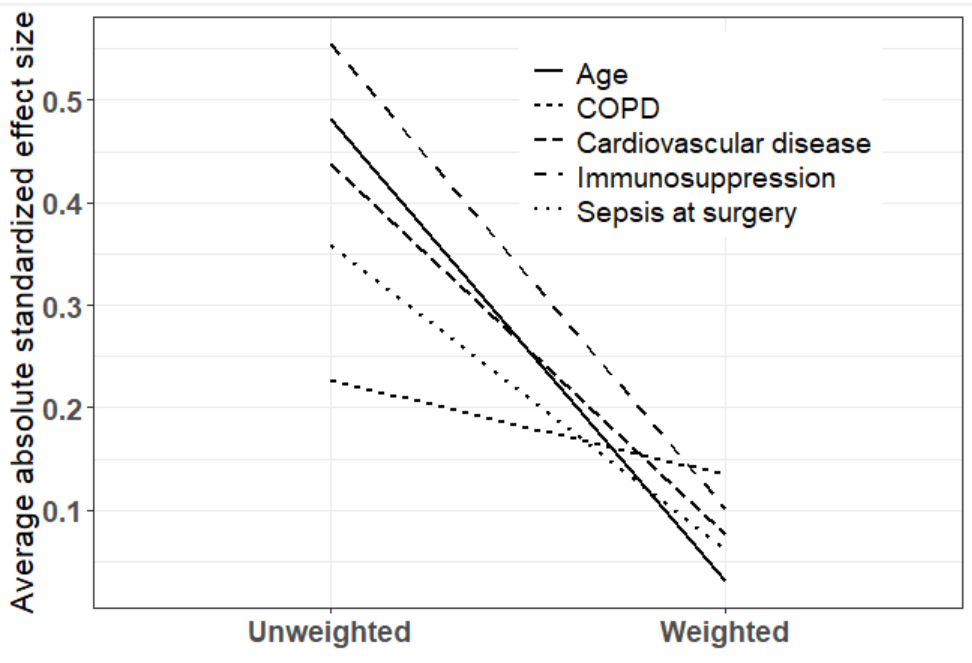


Figure 1. Effect of inverse probability weighting on average absolute standardized effect size

The variability decreases by weighting. Effective Sample Size (ESS) demonstrated how many "effective" patients we have left after weighting. It decreases from 173 to 141 and from 291 to 265 for treatment and control, respectively. This is the price we pay in terms of loss of precision in order to handle the bias due to confounding.

Relative influence on propensity score: Age and immunosuppressive therapy had greatest influence , 56% and 22%, respectively. Smallest influence had COPD (3%).


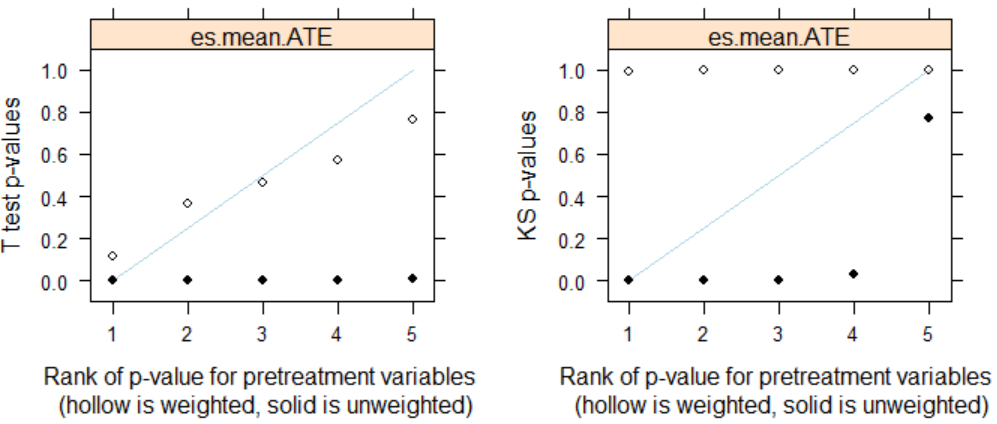


Figure 2. P-values (t-test and Kolomogorov-Smirnov test) of null hypothesis of equality between treatment and control before and after weighting.

Samuelsson A, Bock D, Prytz M, Block M, Ehrencrona C, Wedin A, et al. Laparoscopic lavage for perforated diverticulitis in the LapLav study: population-based registry study. The British journal of surgery. 2021;108(10):1236-42.
